# Research hotpots and frontier trends of neuroprotective effects of magnesium from 1999 to 2023: A bibliometric analysis

**DOI:** 10.1111/cns.14597

**Published:** 2024-02-08

**Authors:** Baoying Song, Miaowen Jiang, Yang Zhang, Yi Xu, Chuanjie Wu, Di Wu, Chen Zhou, Ming Li, Xunming Ji

**Affiliations:** ^1^ Department of Neurology, Xuanwu Hospital Capital Medical University Beijing China; ^2^ China‐America Institute of Neurology, Xuanwu Hospital Capital Medical University Beijing China; ^3^ Beijing Institute for Brain Disorders Capital Medical University Beijing China; ^4^ Department of Neurosurgery, Xuanwu Hospital Capital Medical University Beijing China

**Keywords:** bibliometric analysis, CiteSpace, magnesium, neuroprotective effect, visualization analysis

## Abstract

**Background:**

The neuroprotective effect of magnesium has been widely discussed, and its effectiveness has been confirmed by animal and clinical trials. However, there are still difficulties in clinical translation in diseases such as cerebral ischemia and subarachnoid hemorrhage. Therefore, it is necessary to analyze the literatures about neuroprotection of magnesium to reveal a more comprehensive knowledge framework, research hotspots and trends in the future.

**Methods:**

Original articles and reviews related to neuroprotective effects of magnesium from 1999 to 2022 were retrieved from the Web of Science Core Collection (WoSCC). The bibliometrics CiteSpace 6.2.R4 software was used to conduct co‐occurrence/co‐citation network analysis and plot knowledge visualization maps.

**Results:**

A total of 762 articles from 216 institutions in 64 countries were included in this study. The United States had the largest number of publications, followed by China and Canada. The University of California, UDICE‐French Research Universities, and the University of Adelaide were the top three institutions in publication volume. Crowther Caroline A was the most published and cited author. Among the top 10 cited articles, there were seven articles on neuroprotection in preterm infants and three on acute stroke. Keyword cluster analysis obtained 11 clusters, showing that current research hotspots focused on magnesium therapy in neurovascular diseases such as cerebral ischemia, spinal cord injury, subarachnoid hemorrhage, and emerging treatment strategies.

**Conclusion:**

The neuroprotective effects of magnesium in preterm infants have been extensively studied and adequately confirmed. The therapeutic effects of magnesium on cerebral ischemia and subarachnoid hemorrhage have been demonstrated in animal models. However, the results of clinical studies were not satisfactory, and exploring new therapeutic strategies may be the solution. Recently, the combination of magnesium and hypothermia had great potential in neuroprotective therapy and may become a development trend and hotspot in the future.

## Graphical abstract

Through bibliometric analysis, this study found that the neuroprotective effect of magnesium in cerebral ischemia, subarachnoid hemorrhage, and other fields has been widely concerned, and the combination of magnesium and therapeutic hypothermia may become a development trend and hot spot in the future.

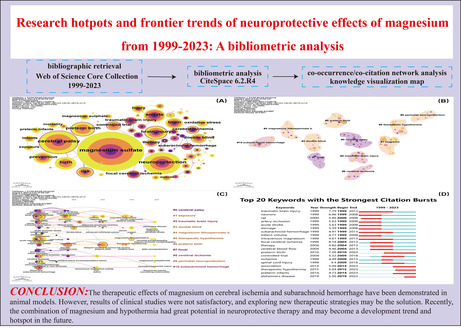

## 1 INTRODUCTION

Magnesium is the fourth most abundant essential mineral in the human body and the second most abundant cation in cells.^1^ Magnesium acts as a coenzyme in more than 300 biochemical reactions, including cellular energy metabolism, protein production, cell growth and reproduction, DNA and RNA synthesis, muscle and nerve function, blood glucose control, and blood pressure regulation.^2^, ^3^, ^4^ Magnesium is distributed about half in bones, half in muscles and other soft tissues, and less than 1% in the blood.^5^ Magnesium also plays an important role in active transport of calcium and potassium ions across cell membranes^6^ and therefore plays a crucial role in nerve impulse conduction, muscle contraction, and maintenance of normal heart rhythm.^7^, ^8^, ^9^ Experimental evidence suggests that magnesium plays a protective role in a variety of neurological disorders, including headache, stress, acute brain injury, stroke, epilepsy, Parkinson's disease, and Alzheimer's disease.^10^

Magnesium exhibits neuroprotective effects through both neuronal and vascular mechanisms. Magnesium is an endogenous antagonist of voltage‐and ligand‐gated calcium channels glutamate‐N‐methyl‐D‐aspartate (NMDA) receptor, which can reduce excitotoxicity caused by calcium overload after nervous system injury and reduce brain cell death.^11^, ^12^ At the same time, magnesium is an important cofactor in energy production process of tricarboxylic acid cycle‐oxidative phosphorylation reaction, which can maintain mitochondrial function and improve energy imbalance after brain injury.^12^, ^13^ Magnesium can also regulate intracellular calcium levels in vascular smooth cells by inhibiting calcium channels.^14^ It reduces the production of endothelin‐1 and inhibits the activation of myosin light chain kinase, thereby attenuating vasospasm and contraction and improving cerebral blood flow.^15^ In addition, studies have shown that magnesium can play a neuroprotective role by regulating blood–brain barrier permeability and reducing inflammatory response.^16^, ^17^ Clinical experiments have demonstrated the neuroprotective efficacy of magnesium in preterm delivery and neonatal hypoxic encephalopathy. Studies on animals have also revealed that magnesium has neuroprotective properties in stroke. However, there are still difficulties in clinical translation in cerebral ischemia, subarachnoid hemorrhage, and other diseases. Therefore, it is necessary to conduct a literature analysis, identify the reasons that hinder its translation, and discover further research trends and hotspots.

In this study, CiteSpace, an information visualization software developed by Professor Chaomei Chen using Java language,^18^ was used to objectively analyze the characteristics of the literatures on magnesium neuroprotection, such as the annual number of publications, countries, institutions, authors, literature citations, and keywords. This analysis can provide a reference for researchers to grasp the research directions and hotspots comprehensively and accurately in neuroprotective effect of magnesium.

## 2 METHODS

### 2.1 Search strategy

The Web of Science Core Collection (WoSCC) was selected as the source database for data retrieval. We searched for relevant studies using the following strategy: TS—(neuroprotection OR neuroprotective) AND TS—(magnesium). The type of literature is limited to “article” or “review,” the language is limited to English and the published time is limited between January 1, 1999, and August 1, 2023, and download in 1 day (August 1, 2023). Excluding publications: book chapter (*n* = 1), proceedings paper (*n* = 24), early access (*n* = 8), and retracted publication (*n* = 1). A total of 762 original English papers (included 570 articles and 192 reviews) were obtained.

### 2.2 Analysis tool

CiteSpace version 6.2.R4 developed by Professor Chaomei Chen was selected to analyze knowledge inflection points, frontier research hotpots, evolutionary directions, and dynamically display the research process and direction of the role of magnesium in neuroprotection. The specific software settings were as follows: Time Slicing was set from January 1999 to August 2023, year per slice was set as five; Node Types were separately selected as country, institution, author, keywords, reference and cited authors; Selection Criteria was set to top *N* (*N* = 50) or g‐index (*k* = 25); Links Strength was selected as cosine; Pruning was selected as pathfinder, sliced networks and merged network.

The output results mainly included the annual publication volume analysis, countries, institutions and authors cooperation analysis, literature and author co‐citation analysis and keywords co‐occurrence, clustering, and burst analysis. The size of the node was positively related to the frequency of occurrence. The purple circle outer ring of the node represented a high mediated centrality (≥0.1), indicating that the node played a crucial connection and pivotal role. The links between nodes represented co‐occurrence relationships, and the thickness of the lines indicates the strength of co‐occurrence. A time line view was used to show the interaction between clusters and the historical span of the literature in the clusters, and the structure and clarity of the views were evaluated using module values (*Q* value) and average silhouette values (*S* value). *Q* value >0.3 indicated high heterogeneity between different clusters, and *S* value >0.7 indicated high consistency within clusters. The log‐likelihood ratio test was used to extract nominal phrases representing the characteristics of a certain cluster study as cluster labels.

## 3 RESULTS

### 3.1 Annual publication trend analysis

**FIGURE 1:**
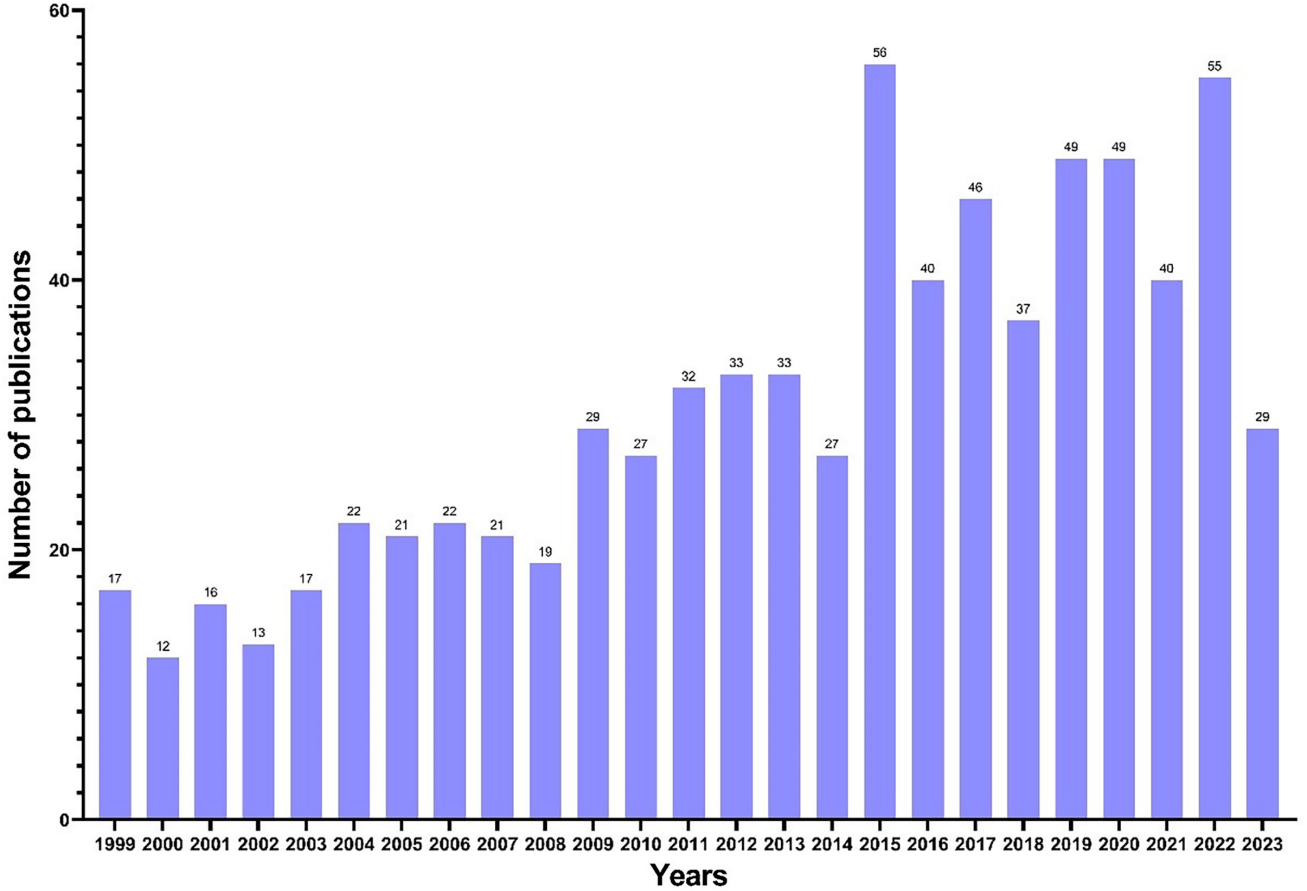
Annual number of published articles from 1999 to 2023.

A total of 762 articles related to the neuroprotection of magnesium published from January 1, 1999, to August 1, 2023, were included in this article. The statistical analysis was carried out according to the publication year, and the annual publication trend is shown in Figure 1. In the past 25 years, the number of publications on neuroprotective effect of magnesium has shown a dynamic upward trend. From 1999 to 2015, the number of published articles showed a slight increasement, and reached its peak in 2015, with 56 articles per year. From 2015 to 2023, the number of documents entered a relatively high level of stability, although with small fluctuations, indicating that the application of magnesium in neuroprotection has attracted more extensive attention. The number of articles published in 2022 reached 55, and although the data in 2023 is still incomplete, it is expected that the final data in 2023 will show a rapid growth trend compared with before.

### 3.2 Countries/regions and institutions cooperation analysis

**FIGURE 2:**
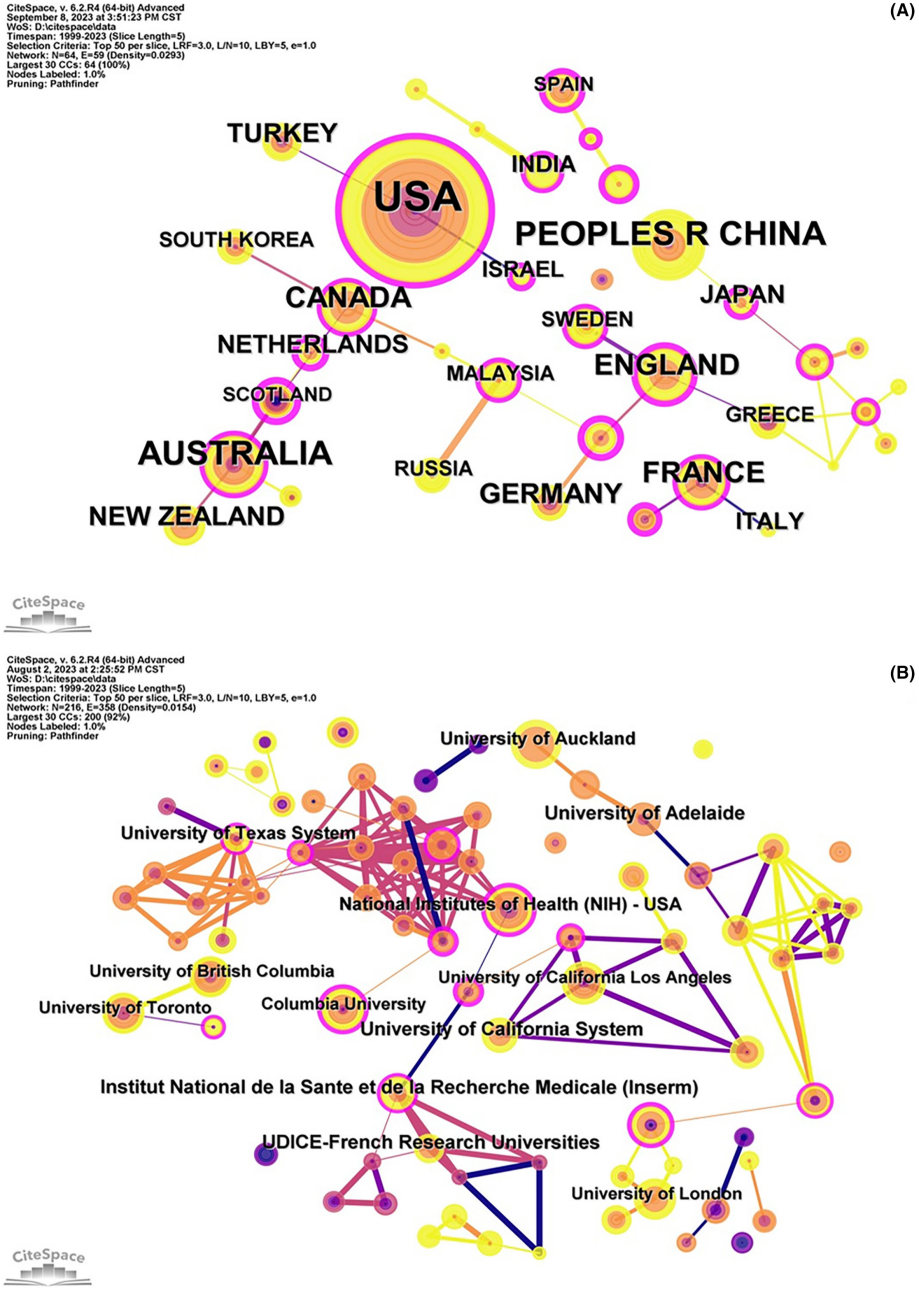
Countries/regions (A) and institutions (B) co‐occurrence network map.

All published articles were from 216 institutions in 64 countries or regions. The co‐occurrence analysis of cooperation network for countries or regions generated a total of 64 nodes and 59 links, with a density of 0.0293 (Figure 2A). As depicted in Table 1, the United States had the largest number of publications (269 publications, 27.12%), followed by China and Australia occupying the second and third place with 80 (8.06%) and 74 (7.46%) publications respectively. Ranked 4–10 are England, Canada, France, Germany, Turkey, New Zealand and the Netherlands. Among the top 10 countries in the number of publications, the top three countries in the mediated centrality were the Netherlands, England and the United States, indicating that the above countries played a constructive mediation role in the international cooperation in exploring magnesium of neuroprotection.

**TABLE 1:** Top 10 countries/regions and top 10 institutions with the most publications.

Rank	Country/Region	Count (%)	Centrality	Institution	Count (%)	Centrality
1	USA	269 (27.12%)	0.25	University of California System	33 (3.02%)	0.04
2	China	80 (8.06%)	0	UDICE‐French Research Universities	30 (2.74%)	0
3	Australia	74 (7.46%)	0.16	University of Adelaide	29 (2.65%)	0.07
4	England	48 (4.84%)	0.28	Institut National de la Sante et de la Recherche Medicale (Inserm)	29 (2.65%)	0.17
5	Canada	47 (4.74%)	0.13	University of Texas System	26 (2.38%)	0.13
6	France	42 (4.23%)	0.16	University of Auckland	25 (2.29%)	0.02
7	Germany	39 (3.93%)	0.05	National Institutes of Health (NIH)—USA	23 (2.10%)	0.39
8	Turkey	33 (3.33%)	0.05	University of California Los Angeles	22 (2.01%)	0.03
9	New Zealand	29 (2.92%)	0	University of Toronto	21 (1.92%)	0.07
10	Netherlands	26 (2.62%)	0.41	University of British Columbia	21 (1.92%)	0

The institutional co‐occurrence network map generated a total of 216 nodes and 358 links, with a density of 0.0154 (Figure 2B). The institution with the largest number of publications was the University of California System (33 publications, 3.02%), followed by the UDICE‐French Research Universities (30 publications, 2.74%), the University of Adelaide (29 publications, 2.65%), the Institut National de la Sante et de la Recherche Medicale (Inserm) (29 publications, 2.65%) and the University of Texas System (26 publications, 2.38%). The United States National Institutes of Health had the highest mediated centrality of 0.39 and ranked seventh in the number of publications, indicating that it was in a crucial position in the cooperation and connection of institutions.

### 3.3 Authors and co‐cited authors analysis

**FIGURE 3:**
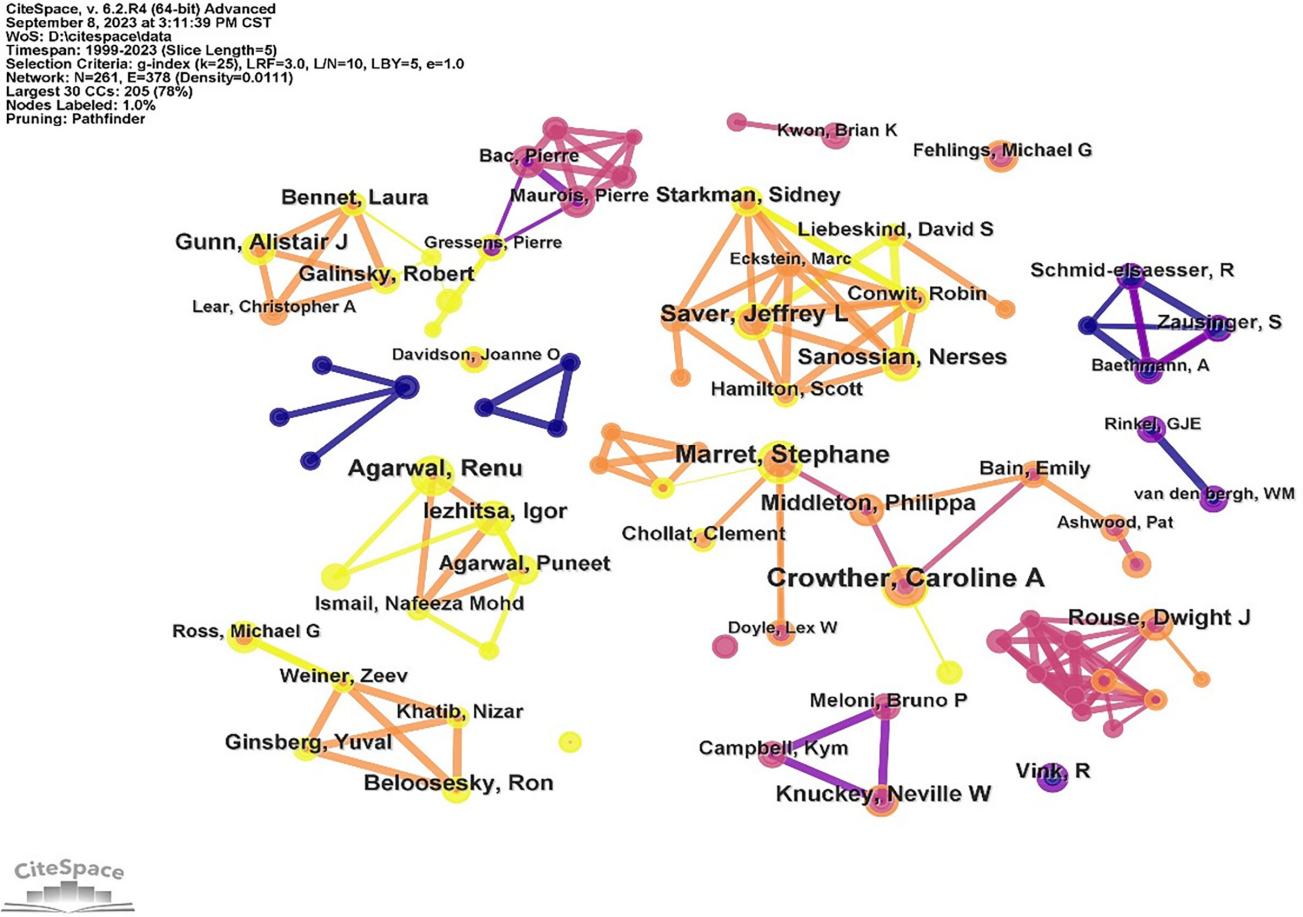
Author collaboration network map.

The authors' co‐occurrence network map generated a total of 261 nodes and 378 links with a density of 0.0111 (Figure 3), indicating that 261 authors who published studies related to magnesium on neuroprotection were included in this analysis. Author analysis was conducted to identify representative scholars and core research forces in this field. Figure 3 shows that there were many research teams, with close cooperation within teams and less cooperation between teams. Among them, Saver Jeffrey L's group had the closest collaboration among different investigators, dedicated to exploring magnesium neuroprotection in stroke. According to Table 2, Crowther Caroline A had the largest number of publications (15 publications, 1.86%), followed by Marret Stephane (14 publications, 1.73%), Saver Jeffrey L (11 publications, 1.36%), and Agarwal Renu (11 publications, 1.36%).

**TABLE 2:** Top 10 authors and co‐authors with the highest publications and citations.

Rank	Author	Count (%)	Co‐author	Citation	Centrality
1	Crowther Caroline A	15 (1.86%)	Crowther Caroline A	184	0.32
2	Marret Stephane	14 (1.73%)	Doyle LW	182	0.13
3	Saver Jeffrey L	11 (1.36%)	Marret Stephane	155	0.45
4	Agarwal Renu	11 (1.36%)	Rouse Dwight J	152	0.02
5	Iezhitsa Igor	10 (1.24%)	Duley L	114	0.1
6	Gunn Alistair J	10 (1.24%)	Nelson Kb	111	0.11
7	Knuckey Neville W	9 (1.11%)	Mittendorf R	111	0.94
8	Rouse Dwight J	9 (1.11%)	Muir Kw	100	0.33
9	Sanossian Nerses	9 (1.11%)	Saver Jeffrey L	85	0.04
10	Beloosesky Ron	9 (1.11%)	Conde‐Agudelo A	85	0.08

Co‐cited authors are defined as at least two authors who were cited simultaneously by at least one article. A total of eight co‐cited authors had their articles cited 100 times. Crowther Caroline A was the most cited author (184) with high mediation centrality (0.32) and was a representative scholar and backbone in neuroprotection by magnesium, to explore the role of magnesium in preventing cerebral palsy in preterm infants and treatment of hypoxic–ischemic encephalopathy.

### 3.4 Co‐cited references analysis

**FIGURE 4:**
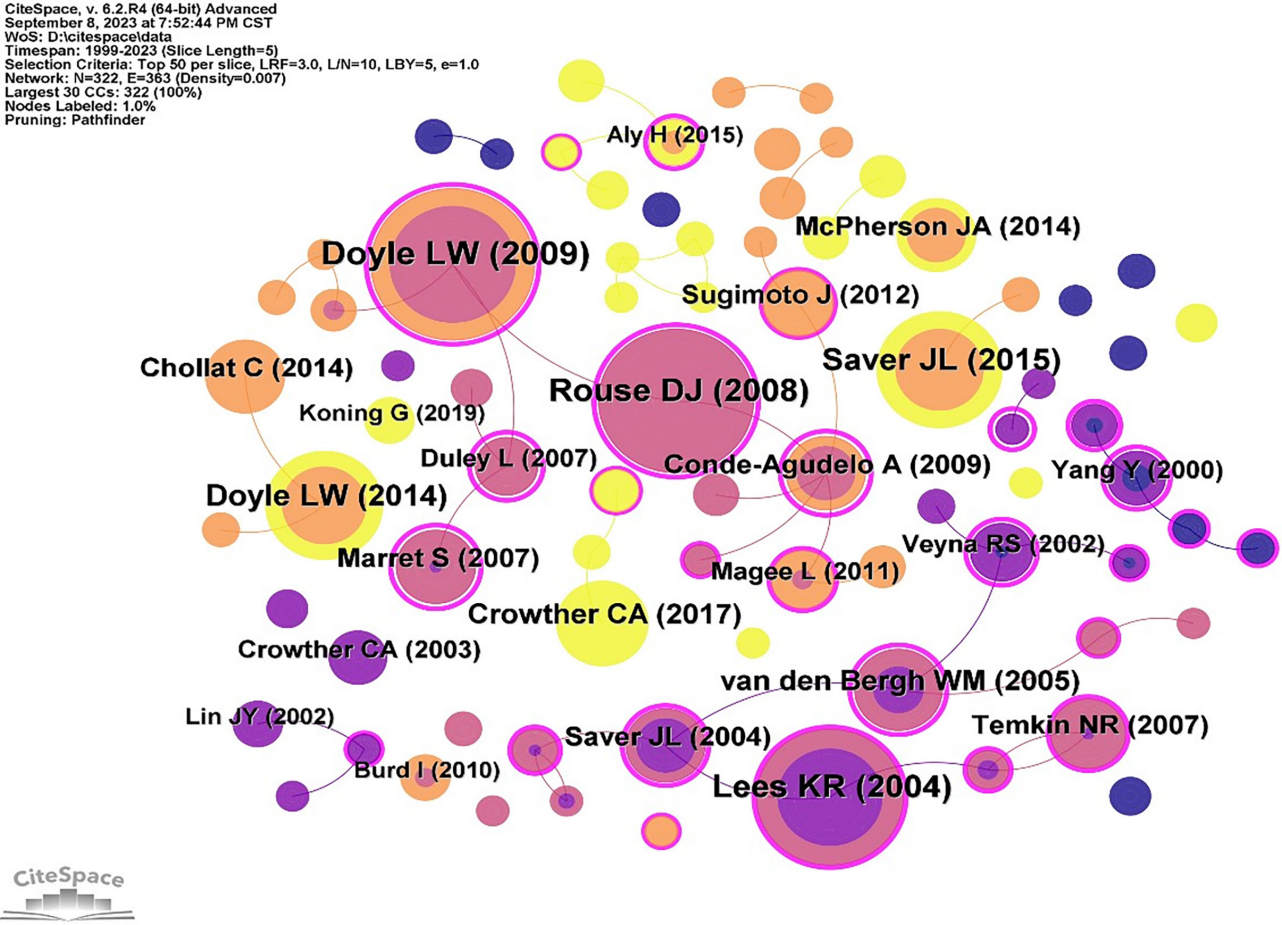
References co‐citation network map.

References co‐cited network map generated a total of 322 nodes and 363 links with a density of 0.007 (Figure 4). The larger the area of the node, the more times the literature has been cited. The literature marked by the purple circle had a higher centrality, which had important reference value and development significance in the research process of this field. The top 10 cited articles are shown in Table 3, with seven articles on neuroprotection in preterm infants and 3 in acute stroke. The effect of magnesium on fetuses at risk of preterm birth neuroprotection has always been a hot topic. The review published by Doyle LW et al. in the “Cochrane Database System Review” entitled “Magnesium sulfate for women at risk of preterm birth for neuroprotection of the fetus” was the most cited article (39 citations) and had a high mediation centrality (0.78). Based on a systematic review of five clinical studies, this study found that prenatal magnesium sulfate treatment of women at risk of preterm birth significantly reduced the risk of cerebral palsy and the rate of substantial gross motor dysfunction in their children.^19^ The second most cited paper was a randomized controlled clinical study by Dwight J Rouse et al., which was published in “The New England Journal of Medicine” in 2008. This multicenter, placebo‐controlled, double‐blind study enrolled 2241 women at risk for preterm birth between 24 and 31 weeks of gestation, and results showed a reduction in the incidence of moderate or severe cerebral palsy in surviving fetuses with magnesium administration.^20^ The first co‐cited reference with the highest centrality was published in “The American Journal of Obstetrics and Gynecology” by Agustín Conde‐Agudelo et al., which conducted a systematic review and meta‐analysis and found that magnesium sulfate administered to women at risk of delivery before 34 weeks of gestation reduced the risk of fetal cerebral palsy^21^

**TABLE 3:** Top 10 co‐cited references.

Rank	Title	Author	Source	Impact factor	Year	Citation	Centrality	DOI
1	Magnesium sulphate for women at risk of preterm birth for neuroprotection of the fetus.	Lex W Doyle, et al.	Cochrane Database Syst Rev	8.4	2009	39	0.78	https://doi.org/10.1002/14651858.CD004661.pub3
2	A randomized, controlled trial of magnesium sulfate for the prevention of cerebral palsy.	Dwight J Rouse, et al.	N Engl J Med	158.5	2008	37	0.75	https://doi.org/10.1056/NEJMoa0801187
3	Magnesium for acute stroke (Intravenous Magnesium Efficacy in Stroke trial): randomized controlled trial.	K W Muir, et al.	Lancet	168.9	2004	35	0.27	https://doi.org/10.1016/S0140‐6736(04)15490‐1
4	Prehospital use of magnesium sulfate as neuroprotection in acute stroke.	Jeffrey L Saver, et al.	N Engl J Med	158.5	2015	30	0.02	https://doi.org/10.1056/NEJMoa1408827
5	School‐age outcomes of very preterm infants after antenatal treatment with magnesium sulfate vs placebo.	Lex W Doyle, et al.	JAMA	120.7	2014	28	0.05	https://doi.org/10.1001/jama.2014.11189
6	Assessing the neuroprotective benefits for babies of antenatal magnesium sulphate: An individual participant data meta‐analysis.	Caroline A Crowther, et al.	PLoS Med	15.8	2017	22	0.08	https://doi.org/10.1371/journal.pmed.1002398
7	Magnesium sulfate in aneurysmal subarachnoid hemorrhage: a randomized controlled trial.	Walter M van den Bergh, et al.	Stroke	8.3	2005	21	0.7	https://doi.org/10.1161/01.STR.0000160801.96998.57
8	School‐age outcomes following a randomized controlled trial of magnesium sulfate for neuroprotection of preterm infants.	Clément Chollat, et al.	J Pediatr	5.1	2014	19	0	https://doi.org/10.1016/j.jpeds.2014.04.007
9	Antenatal magnesium sulfate for the prevention of cerebral palsy in preterm infants less than 34 weeks' gestation: a systematic review and meta‐analysis.	Agustín Conde‐Agudelo, et al.	Am J Obstet Gynecol	9.8	2009	19	0.89	https://doi.org/10.1016/j.ajog.2009.04.005
10	Magnesium sulphate given before very‐preterm birth to protect infant brain: the randomized controlled PREMAG trial.	S Marret, et al.	BJOG	9.8	2007	19	0.84	https://doi.org/10.1111/j.1471‐0528.2006.01162.x

Another important research direction is magnesium for neuroprotection in stroke. The third most cited article was published by K W Muir et al. in the Lancet in 2004. This randomized controlled trial known as the IMAGES study was one of the largest trials of a putative neuroprotective agent at the time and was well‐designed. Results of the analysis of 2589 patients with acute stroke showed that intravenous magnesium administration within 12 h after acute stroke did not significantly reduce the chance of death or disability.^22^ The fourth most cited study by Jeffrey L Saver et al. advanced the use of magnesium in the prehospital setting for acute stroke. Of 1700 patients, 857 received magnesium injections and the rest received a placebo, which was initiated within 2 h of the onset of stroke symptoms. The results showed that prehospital treatment with magnesium sulfate was safe but did not improve clinical outcomes at 90 days in stroke patients.^23^

### 3.5 Keywords co‐occurrence, clustering, timeline and burst analysis

**FIGURE 5:**
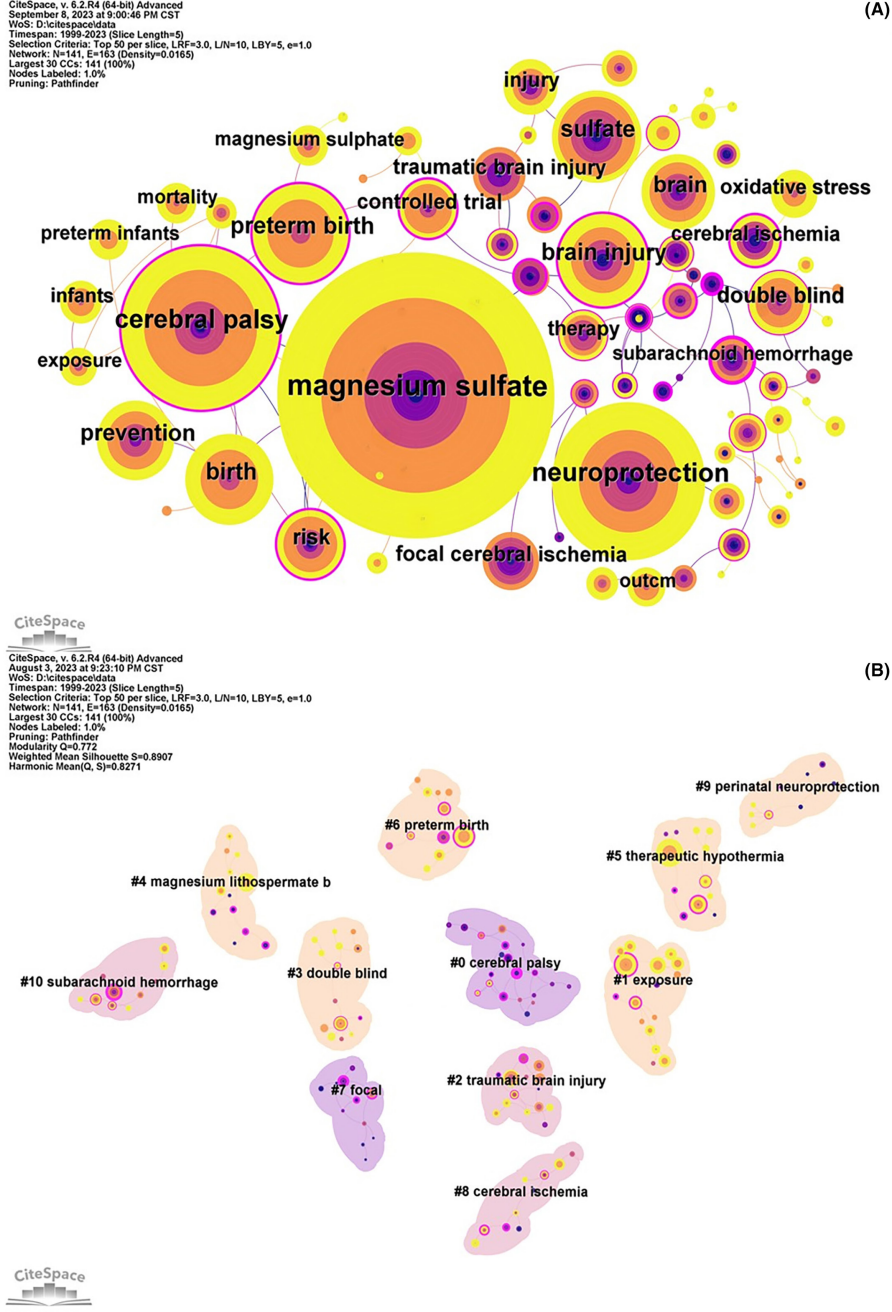
Network diagram of keywords co‐occurrence (A) and keywords cluster map (B).

The analysis of keywords can observe the dynamic research hotspots and trends in this field. The keyword co‐occurrence map showed a total of 141 nodes, 163 links, with a density of 0.0165 (Figure 5A). The top 20 keywords with frequency are shown in Table 4. The diseases or applications related to the neuroprotective effect of magnesium include cerebral palsy, preterm birth, brain injury, cerebral ischemia, traumatic brain injury, and subarachnoid hemorrhage. The research directions mainly included prevention, risk, blinding, controlled trial and treatment, and the hot research mechanism was oxidative stress.

**TABLE 4:** Top 20 co‐occurrence keywords.

Rank	Keyword	Occurrences	Centrality	Rank	Keyword	Occurrences	Centrality
1	Magnesium sulfate	226	0.06	11	Double blind	50	0.12
2	Cerebral palsy	129	0.34	12	Controlled trial	46	0.27
3	Neuroprotection	125	0.05	13	Focal cerebral ischemia	46	0.03
4	Preterm birth	78	0.21	14	Injury	44	0.04
5	Brain injury	73	0.24	15	Traumatic brain injury	43	0.05
6	Sulfate	73	0.05	16	Cerebral ischemia	40	0.23
7	Birth	72	0.09	17	Therapy	39	0.19
8	Prevention	63	0	18	Oxidative stress	38	0
9	Brain	60	0.03	19	Infants	34	0
10	Risk	54	0.39	10	Subarachnoid hemorrhage	33	0.43

**FIGURE 6:**
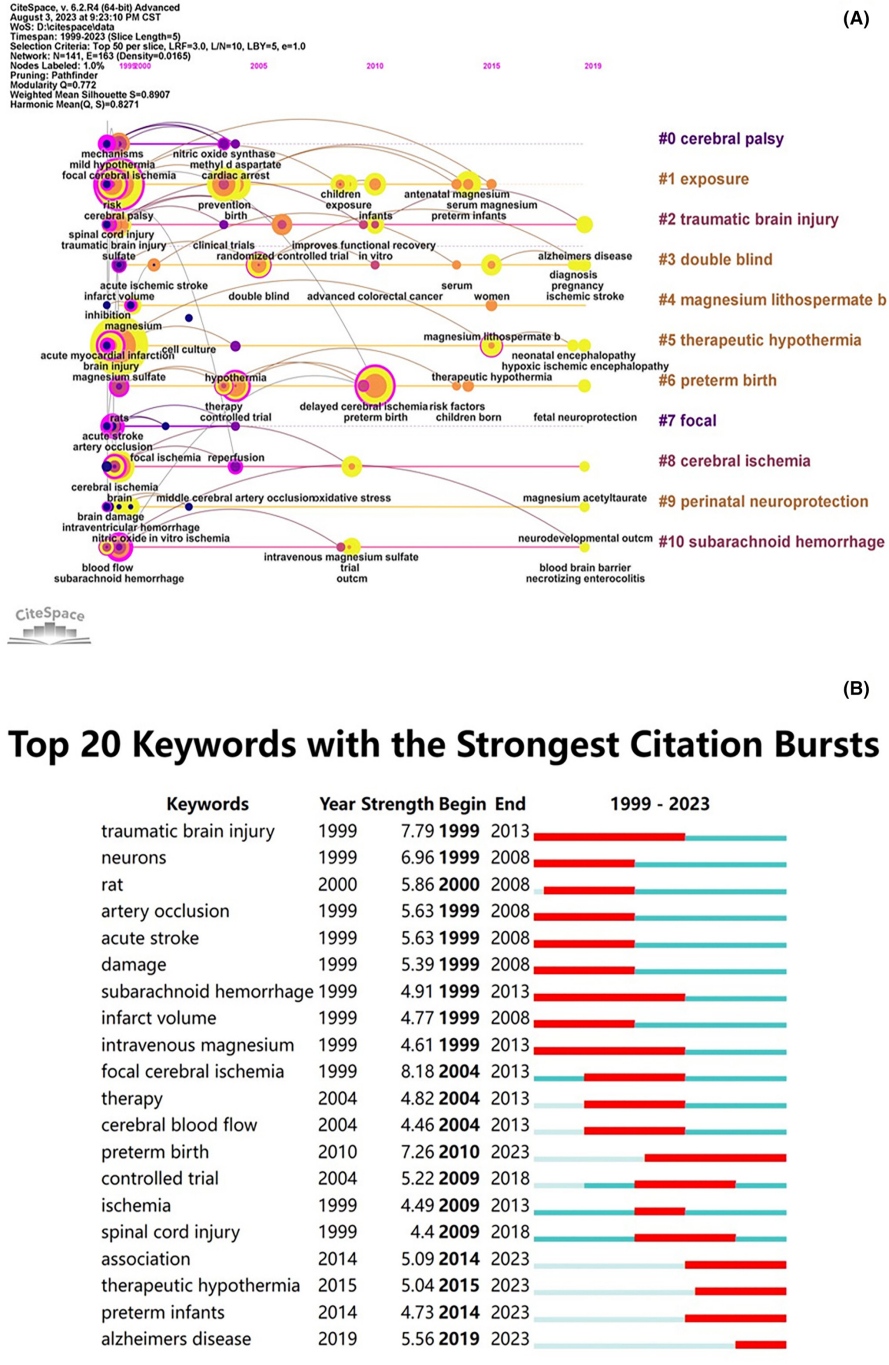
Network diagram of keywords timelines (A) and top 20 keywords with the strongest citation bursts (B).

The log‐likelihood ratio algorithm was used to cluster keywords, and the feature labels were used to identify and rank each cluster to determine the research frontiers (Figure 5B). The overall module value of clustering *Q* = 0.772 (>0.3) indicated that the cluster structure was significant, and there was high heterogeneity among different clusters. A total of 11 clusters were generated, and the silhouette values of each cluster were above 0.7, indicating a high consistency within the cluster. According to the cluster structure, they were labeled from large to small from #0 to #10: #0 cerebral palsy, #1 exposure, #2 traumatic brain injury, #3 double‐blind, #4 magnesium lithospermate b; #5 therapeutic hypothermia; #6 preterm birth, #7 focal, #8 cerebral ischemia; #9 perinatal neuroprotection, #10 subarachnoid hemorrhage (Table 5). In the timeline diagram, keywords of the same cluster were arranged in chronological order of first appearance (Figure 6A), demonstrating the development of research on neural protective effects of magnesium.

**TABLE 5:** Keywords cluster analysis.

Cluster‐ID	Size	Silhouette	Mean year	Coverage	Label
0	20	0.774	2000	Cerebral palsy; mild hypothermia; fast muscle; central nervous system ischemia; channels	Cerebral palsy
1	16	1	2007	Cerebral pals; exposure; mortality; birth; preterm delivery	Exposure
2	14	0.918	2003	Traumatic brain injury; spinal cord injury; polyethylene glycol; magnesium; cerebral palsy	Traumatic brain injury
3	14	0.804	2009	Double blind; cerebral palsy; cisplatin; ischemic stroke; chemotherapy	Double blind
4	12	0.884	2002	Magnesium lithospermate b; neurotoxicity; er stress; activation; cell culture	Magnesium lithospermate b
5	12	0.943	2007	Therapeutic hypothermia; brain injury; hypoxic–ischemic encephalopathy; hypoxic ischemic encephalopathy; neonatal encephalopathy	Therapeutic hypothermia
6	11	0.856	2010	Preterm birth; magnesium sulphate; controlled trial; cerebral palsy; implementation	Preterm birth
7	11	0.956	1999	Focal; drug therapy; cerebral ischemia; rats; combination	Focal
8	10	0.932	2003	Cerebral ischemia; cerebral palsy; excitotoxicity; glaucoma; taurine	Cerebral ischemia
9	10	0.927	2003	Perinatal neuroprotection; intrauterine infection; fetus; birth weight infants; antenatal magnesium sulfate	Perinatal neuroprotection
10	10	0.953	2008	Subarachnoid hemorrhage; vasospasm; cerebrospinal fluid; delayed cerebral ischemia; brain edema	Subarachnoid hemorrhage

CiteSpace provides the burst detection function to explore large changes in citation to predict the migration and change of research frontiers. The top 20 keywords with the strongest citation bursts are shown in Figure 6B. From 1999 to 2008, magnesium treatment for traumatic brain injury, arterial occlusion, acute stroke, and subarachnoid hemorrhage attracted the attention of researchers. The main research methods were basic experiments in rats and intravenous magnesium, and the main directions were mechanism research including neurons, injury and infarct volume. Since 2010, more attention has been focused on magnesium in preterm birth, leading to a rapid development of the knowledge framework and clinical application in this field. At the same time, research on neuroprotection in ischemic encephalopathy was also ongoing but progress was relatively slow. In recent years, researchers have focused on the association between magnesium and neurological diseases such as ischemia, spinal cord injury, Alzheimer's disease, and the exploration of the combination of therapeutic hypothermia. These emerging keywords represented the future research trends and hotspots in this field.

## 4 DISCUSSION

Our study included relevant literatures in the field of magnesium neuroprotective effects from 1999 to 2023. Based on the above bibliometric analysis, we provided a detailed review of the main research areas of the neuroprotective effects of magnesium, including magnesium therapy in perinatal, ischemic encephalopathy, subarachnoid hemorrhage, and emerging treatment modalities and strategies.

### 4.1 Magnesium therapy for perinatal neural protection

Preterm birth is one of the most important causes of increased risk of perinatal neonatal death^24^ and is associated with severe adverse neurological outcomes such as cerebral palsy, motor impairment, blindness, deafness, developmental delay, cognitive delay, poor academic performance, and behavioral disorders.^25^ According to the analysis of keywords emergence from CiteSpace, neural protective effect of magnesium in premature infants caused widespread attention since 2010, which might be related to several important clinical studies conducted and results published. The ACTOMgSO_4_ trial by Caroline A Crowther et al. treated women at 30 weeks of gestation who were at risk for acute preterm birth with magnesium sulfate. Two years of follow‐up results showed that substantial gross motor dysfunction, and combined death or substantial gross motor dysfunction were significantly reduced.^26^ In the study of French PREMAG, administration of magnesium sulfate to women with a gestational age of less than 33 weeks who were expected to deliver within 24 h resulted in a reduction in total fetal mortality and rates of severe white‐matter injury.^27^ In BEAM trial, Magnesium treatment of women at risk for preterm birth between 24 and 31 weeks of gestation significantly reduced the frequency of moderate or severe fetal cerebral palsy.^20^ In the subsequent study, in which survivors from ACTOMgSO_4_ and PREMAG trials participated in school‐age follow‐up, children in the magnesium group showed no significant differences in neurologic, cognitive, behavioral, growth, or functional outcomes from those in the control group, but superiority in the risk of death cannot be excluded.^28^, ^29^ Based on a secondary analysis of BEAM data, magnesium sulfate administration continued over 18 h, was given within 12 h after delivery, and maintained maternal serum magnesium levels of 4.1 mg/dL, maximizing the neuroprotective effects of the drug.^30^, ^31^, ^32^, ^33^

### 4.2 Magnesium therapy for cerebral ischemic diseases

Cerebral ischemic disease is the most common cerebrovascular disease, and its high mortality and disability seriously damage human health. The safety of magnesium therapy has been widely confirmed in premature infants, eclampsia, and neonatal hypoxic–ischemic encephalopathy, it has been gradually applied to the neuroprotective treatment of cerebral ischemic diseases. As shown in the CiteSpace analysis, from 1999 to 2008, the research of magnesium of cerebral ischemia received extensive attention. Animal studies have shown that intraperitoneal magnesium supplementation at 1 h or 30 min before ischemia can reduce the volume of cerebral infarction in rats with focal cerebral ischemia caused by middle cerebral artery occlusion.^12^, ^34^ Moreover, intraperitoneal magnesium supplementation could improve the ischemic injury of global cerebral ischemia model by reducing the apoptosis of neurons.^35^ Studies indicated that serum magnesium concentration of 2.0–2.5 mmol/L had the best neuroprotective effect and intravenous magnesium supplementation further increased plasma magnesium concentration and reduced the influence of first‐pass effect.^36^ Early magnesium treatment (within 2 h after ischemia) increased the survival rates, reduced the infarct volume and improved neurological function in rats with cerebral ischemia.^37^, ^38^ Intraarterial magnesium supplementation rapidly increased the concentration of magnesium in the ischemic area and attenuated cardiac inhibition through the systemic circulation. Pre‐ischemia administration of magnesium sulfate through the carotid artery significantly reduced infarct volume in focal ischemia model rats.^39^, ^40^ However, all these pathways were blocked by the blood–brain barrier, thus decreasing the concentration of magnesium entering the brain tissue. Subsequent trials of intracranial magnesium therapy discovered that parenchymal magnesium supplementation within 24 h after ischemia reduced nerve death in the ischemic brain region.^41^, ^42^

Based on the promising effects in animal ischemic models, a series of clinical trials of magnesium as a neuroprotective agent in the treatment of cerebral ischemic diseases have been carried out (Table 6). The clinical studies had adequately confirmed the safety of magnesium but did not achieve significant improvement in clinical outcomes. In a substudy of IMAGES research, through the use of magnetic resonance imaging, with the growth of the cerebral infarction imaging results replacing the progression of clinical neurological symptoms, the therapeutic effect of magnesium was analyzed. The results demonstrated no significant differences in infarct growth between magnesium treatment group and placebo group, which was consistent with the clinical outcomes.^43^ Subgroup analysis of the IMAGES study suggested that magnesium might have an improved outcome in patients with lacunar infarction.^22^ A subsequent analysis of IMAGES data with adjustment for stratification variables provided further evidence of the positive effect of magnesium on lacunar infarction, however, further clinical studies were needed to confirm this finding.^44^ The negative results of these major clinical studies might explain the gradual decline in research heat on magnesium in ischemic cerebrovascular disease since 2013. Researchers hypothesized about possible reasons for negative results. In IMAGES study, mean duration of magnesium supplementation was 7 h, and only 3% was administered within 3 h after onset, which resulted in a decrease in volume of salvageable brain tissue, thus affecting clinical effect. In FAST‐MAG trial, although mean duration of intravenous magnesium supplementation in prehospital setting was reduced to 45 min and serum magnesium concentration reached a threshold for a protective effect, it was unknown whether magnesium increase in brain tissue would have a beneficial effect. An exploratory analysis of ultra‐early neurodegeneration in patients with acute cerebrovascular disease from FAST‐MAG study showed that ultra‐early neurological deterioration was significantly associated with poor outcomes and increased mortality.^45^ These results suggested that initiating stroke treatment as early as possible was advisable. Therefore, magnesium supplementation might still be administered early and achieve a brain‐protective dose as soon as possible.

**TABLE 6:** Summary of clinical trials of magnesium in stroke.

Author	Stroke type	Sample size: Magnesium/Placebo	Time administered (hour/h)	Dose	Primary outcome measure	Conclusion
Keith W. Muir et al.^79^	Ischemia 100%	30/30	Within 12 h	8 mmol IV over 15 min and 65 mmol over 24 h	Barthel Index score < 60 at 90 days	Magnesium well tolerated, with no significant side‐effects.
Keith W. Muir et al.^80^	Ischemia 100%	19/6	Within 24 h	8, 12, or 16 mmol IV over 15 min and 65 mmol over 24 h	Barthel Index score and Rankin Scale score at 30 and 90 days	Magnesium well tolerated, with 16 mmol loading infusion chosen for further trials.
Yair Lampl et al.^81^	Ischemia 100%	22/19	Within 24 h	4 g IV over 15 min and 35 g over 24 h for 5 days	Barthel Index score and Rankin Scale score at 30 days	Intravenous magnesium sulfate had significant positive effect on outcome in patients with acute stroke.
IMAGES trial.^22^	Ischemia 92%	1188/1198	Within 12 h	16 mmol IV over 15 min and 65 mmol over 24 h	Common odds ratio for death or disability at 90 days	Magnesium did not reduce the chances of death or disability significantly in acute stroke, although might be benefit in lacunar strokes.
FAST‐MAG pilot trial.^82^	Ischemic 80%; Hemorrhagic 20%	20/25	Within 12 h	2.5 g IV over 10 min, 1.5 g in emergency and 16 g over 24 h	Modified Rankin scale at 90 days	Field initiation of Mg sulfate in acute stroke patients is feasible and safe.
FAST‐MAG trial.^23^	Ischemia 73.3%; Hemorrhage 22.8%	857/843	Within 2 h	Mean 45 min after symptom onset; 4 g IV over 15 min and 16 g over 24 h	Modified Rankin scale at 90 days	Prehospital initiation of magnesium sulfate therapy was safe, but did not improve disability outcomes at 90 days.

Abbreviation: IV, intravenous injection.

### 4.3 Magnesium therapy for subarachnoid hemorrhage

Although results of CiteSpace analysis suggested that researches on magnesium therapy in subarachnoid hemorrhage gradually decreased after 2013, we still analyzed this field for the following reasons. One reason was that management of aneurysmal subarachnoid hemorrhage had improved considerably with development of surgery and endovascular treatment. However, delayed cerebral ischemia (DCI) due to cerebral vasospasm after aneurysm ablation remains one of the most serious complications leading to death or neurologic deterioration.^46^, ^47^, ^48^ Another reason was that magnesium had been studied as a pre‐hospital emergency treatment for acute stroke, and subarachnoid hemorrhage is an important subtype of acute stroke; hence, magnesium neural protective effect of subarachnoid hemorrhage is worth further discussing. In rat model, intravenous magnesium supplementation reversed cerebral vasospasm and reduced brain lesion volume in acute phase after subarachnoid hemorrhage.^49^, ^50^ Preclinical trials of intracisternal magnesium infusion in animal models of experimental subarachnoid hemorrhage showed significant dilation of spastic cerebral vessels and improvement reduction in cerebral blood flow.^51^, ^52^, ^53^, ^54^

The safety and efficacy of magnesium in treatment of vasospasm and delayed cerebral ischemia after subarachnoid hemorrhage was initially demonstrated in animal studies, series of clinical trials were subsequently conducted. Several small randomized clinical trials suggested that magnesium administration was safe after subarachnoid hemorrhage, and seemed to be effective in reducing delayed cerebral hemorrhage and improving poor prognosis. However, results did not show significant statistical significance, and a large sample of clinical research is still needed to further confirm its effects.^55^, ^56^, ^57^, ^58^, ^59^ Contrary to expectations, IMASH trial involving 327 patients and MASH‐2 trial involving 1024 patients demonstrated that intravenous magnesium sulfate therapy did not improve the clinical outcome of patients with subarachnoid hemorrhage.^60^, ^61^ A meta‐analysis of 13 trials collected analogous data in 2401 patients with subarachnoid hemorrhage found that prophylactic intravenous magnesium did not improve neurological outcomes or reduce cerebral infarction or mortality.^62^ One of the reasons for lack of effective clinical outcomes for magnesium might be delay in administration of the drug. In a meta‐analysis, only 83 of 1965 patients were treated within 6 h, and small subgroups made it difficult to analyze the effect of dosing time windows on treatment effects.^63^ Moreover, cerebral vasospasm was not the only mechanism of delayed cerebral infarction,^64^ and magnesium treatment alone might be arduous to achieve a desirable effect. Combination therapy of multiple mechanisms may be a promising solution.

### 4.4 Magnesium combined with therapeutic hypothermia strategy

According to burst word analysis of CiteSpace, research on therapeutic hypothermia in neuroprotection of magnesium has attracted extensive attention since 2015. The neuroprotective mechanism of therapeutic hypothermia has been widely researched and affects physiological progress at multiple stages after cerebral ischemia. It can play a regulatory role through multiple pathways such as apoptosis, excitotoxicity, inflammatory response, cell metabolism, blood–brain barrier, and blood flow.^65^ The neuroprotective effect of hypothermia on patients with cardiac arrest and neonatal hypoxic–ischemic encephalopathy has been confirmed, and both of them have been recommended as a feasible treatment by clinical practice guidelines.^66^, ^67^ In recent years, clinical studies have combined local hypothermia with vascular recanalization therapy, which has been confirmed feasibly and safely in acute stroke.^68^, ^69^

As a neuroprotective agent, magnesium has a synergistic effect when combined with therapeutic hypothermia, thus exerting a more powerful effect. In focal or global cerebral ischemia models, magnesium supplementation combined with hypothermia had a better neuroprotective effect than magnesium alone (Table 7). It may also explain one of the reasons why effective results have not been achieved in clinical trials of magnesium treatment in stroke patients. Because clinical management of stroke requires maintenance of normal body temperature, which probably affects neuroprotective effect of magnesium. Although clinical studies of magnesium combined with hypothermia have not been conducted, the safety and efficacy of animal studies suggested that it is a promising clinical treatment strategy. In recent years, magnesium sulfate combined with therapeutic hypothermia in management of neonatal hypoxic–ischemic encephalopathy is being studied, and large‐scale randomized controlled trials are still needed to further explore.^70^, ^71^

**TABLE 7:** Neuroprotective effects of magnesium combined with hypothermia.

Authors	Animal model	Administration route and method of cooling	Infarct time	Administration time and dose	Temperature and duration	Result
R Schmid‐Elsaesser et al.^83^	tMCAO SD rats	Intravenous; Surface cooling	90 min	Before ischemia and at reperfusion; MgCl_2_ 1 mmol/kg	33°C; 20 min before ischemia until 30 after reperfusion	Efficacy of mild hypothermia (33°C) can be increased by combination pharmacotherapy with tirilazad and magnesium.
Stefan Zausinger et al.^84^	tMCAO SD rats	Intravenous; Surface cooling	90 min	Before ischemia and at reperfusion; MgCl_2_ 1 mmol/kg	33°C; 20 min before ischemia until 30 after reperfusion	Combination therapy with MTH reduced total infarction by 73% and almost completely abolished cortical infarction (−91%).
Stefan Zausinger et al.^85^	tMCAO SD rats	Intravenous; Surface cooling	90 min	Immediately before and 1,3,5 h after ischemia; MgCl_2_ 1 mmol/kg	33°C; 2 h	Therapeutic window of combination therapy with MTH is at least 3 h after onset of ischemia.
Hongdong Zhu et al.^86^	Global cerebral ischemia SD rats	Intravenous; Body temperature allowed to self‐regulate	8 min	Immediately before ischemia; MgSO_4_ 360 μmol/kg	35.4–37.8°C; 6 h	Only the combination of MgSO_4_ treatment and post‐ischemic mild hypothermia is neuroprotective following global ischemia.
Hongdong Zhu et al.^87^	global cerebral ischemia SD rats	Intravenous; Surface cooling	8 min	2 h before and after ischemia; MgSO_4_ 360 μmol/kg	35 ± 0.5°C; 6,12,24 h	Post‐ischemic treatment with a 24 h duration of modest hypothermia and magnesium is more effective than either treatment used alone.
Kym Campbell et al.^88^	tMCAO SD rats	Intravenous; Body temperature allowed to self‐regulate	45 min	Immediately before ischemia; MgSO_4_ 360 or 720 μmol/kg	36.3 ± 0.2°C; 0.5–3 h	Mild spontaneous hypothermia contributed to the observed neuroprotective effect of magnesium.
Kym Campbell et al.^89^	pMCAO SD rats	Intraarterial; Surface cooling	N/A	2,4,6 h after ischemia; MgSO_4_ 360 μmol/kg	35°C; 24 h	Combined MgSO_4_ and hypothermia treatment reduced infarct volumes by 54% at 2 h and by 39% at 4 h, but not 6 h.
Kym Campbell et al.^90^	tMCAO SH SD rats	Intravenous; Surface cooling	90 min	30 min post‐reperfusion; MgSO_4_ 360 μmol/kg	33 or 35°C; 24 h	Mild hypothermia plus magnesium significantly reduced infarct volume.
Wei Song et al.^76^	tMCAO SD rats	Intra‐carotid; intra‐carotid cold infusion	180 min	Before reperfusion; MgSO_4_ 120 mg/kg	33–34°C; 20 min	Local hypothermia induced by intra‐carotid administration of cold magnesium is more effective in reducing acute ischemic damage than infusion of cold saline alone.

Abbreviations: pMCAO, permanent middle cerebral artery occlusion; SD, Sprague–Dawley; SH, spontaneous hypertension; tMCAO, transient middle cerebral artery occlusion.

### 4.5 Future therapeutic strategies for magnesium as neuroprotective agent

The neural protective effects of magnesium have been demonstrated in several animal models of cerebrovascular disease, and it has been recommended in clinical management of preterm infants with hypoxic–ischemic encephalopathy and cardiac arrest patients. However, its clinical efficacy in stroke has not been proven. Future directions will focus on finding effective delivery routes, dosage forms, and combination treatment strategies for magnesium as a neuroprotective agent.

Both intravenous and intraarterial magnesium supplementation were hampered by the blood–brain barrier. Although studies had shown that BBB integrity was disrupted after stroke, an increase in BBB permeability was a stepwise process that required reduction in blood flow that lasted for several hours.^72^ The results of clinical studies suggested that magnesium supplementation as early as possible may improve clinical prognosis of stroke patients, and so intracranial magnesium may become a feasible administration route. Intracisternal magnesium sulfate has been attempted in subarachnoid hemorrhage, and results showed that it could reduce incidence of cerebral vasospasm and delayed cerebral ischemia and improve clinical outcomes.^73^ In cerebral ischemia, although routine surgery is not required, minimally invasive procedures can still be used to explore cisternal or intraventricular magnesium.

Although FAST‐MAG trial, which explored magnesium as a pre‐hospital emergency drug for acute stroke, did not achieve beneficial results, it provided a valuable evaluation and disposal method for magnesium application in pre‐hospital treatment. It is worth thinking about how to combine convenience of drug administration with rapid achievement of effective doses. Oral high‐permeability magnesium compound, currently known as magnesium L‐threonine, has been shown to increase Mg^+^ concentration in cerebrospinal fluid (CSF) in Parkinson's and Alzheimer's disease mouse models, with neuroprotective effects and cognitive function improvement.^74^, ^75^ Its research value in cerebrovascular disease deserves further exploration.

Limited by the time window of thrombolytic therapy, a considerable number of cerebral ischemia patients require endovascular treatment. Selective intra‐arterial hypothermia after recanalization has a clinical benefit trend in patients with intracranial large vessel occlusion. In a rat model of transient middle cerebral artery occlusion, Wei Song et al. found that local intra‐carotid instillation of cold magnesium sulfate was more efficient in reducing acute ischemic injury than instillation of cold saline alone.^76^ Both magnesium and hypothermia can exert neuroprotective effects through multiple targets, which may partially explain their abilities to play a synergistic role. The results suggested that local brain hypothermia combined with magnesium could provide neuroprotection during the critical early stages of cerebral ischemia, extend the time window for thrombolytic therapy, and help reduce the occurrence of reperfusion injury. The concern is whether the injection of cold fluid into the local carotid artery affects hemodilution, which may lead to adverse consequences. However, clinical studies have found that the body can cope with rapid intravenous infusion of high‐volume (>2000 mL/h) cold saline, and the fluid volume and infusion speed that required for local cerebral hypothermia to achieve the target temperature are much smaller than the maximum range.^77^, ^78^ Thus, intra‐carotid infusion of hypothermia magnesium solution after mechanical recanalization may be a new treatment strategy for patients with acute ischemic stroke who are eligible for endovascular treatment.

## 5 LIMITATION

To our knowledge, this is the first bibliometric study of articles in neuroprotective effect of magnesium using co‐occurrence analysis and visual display of CiteSpace software. However, there are several limitations to our study. First, our study only included relevant articles published in the WoSCC database from 1999 to 2023, due to limitations of the software and the database itself. In addition, to facilitate analysis and generalization, we restricted the language to English and included only original articles and reviews to avoid duplication. Therefore, the data may not be comprehensive. We have also included articles outside restrictions for supplementary explanations in subsequent discussion section to elaborate on developments and trends in the field of magnesium neuroprotection as logically and clearly as possible.

## 6 CONCLUSION

This study uses an objective and quantitative method to explore the current status of magnesium neuroprotection in the past 25 years, which aims to provide researchers with a comprehensive and macro perspective to show the development trend of this field. Visual analysis demonstrated that this field of study is rapidly developing, with a consistent growth trend shown in the continually growing linked literature on the subject. The research focuses on preterm infant hypoxic encephalopathy, cerebrovascular disease, cognitive disorders and other neurological diseases. The neuroprotective effect of magnesium on preterm infants has been well‐established and recommended by clinical guidelines. However, the clinical translations of magnesium in cerebral ischemia and subarachnoid hemorrhage are still problems that perplex researchers. The combination of magnesium and hypothermia has shown good potential and may become a development tendency and hot spot in the future.

## AUTHOR CONTRIBUTIONS

Baoying Song, Miaowen Jiang, Xunming Ji, and Ming Li raised the conception of the study and designed the study. Baoying Song conducted the CiteSpace analysis, screened articles and wrote the original manuscript. Yang Zhang, Yi Xu, Chuanjie Wu, Di Wu, and Chen Zhou revised the manuscript and edited critically. All authors contributed to the article and approved the submitted version.

## CONFLICT OF INTEREST STATEMENT

The authors declare no competing interests.

## Data Availability

The data that support the findings of this study are available from the corresponding author upon reasonable request.
